# Expatriates’ Multiple Fears, from Terrorism to Working Conditions: Development of a Model

**DOI:** 10.3389/fpsyg.2016.01571

**Published:** 2016-10-13

**Authors:** Gabriele Giorgi, Francesco Montani, Javier Fiz-Perez, Giulio Arcangeli, Nicola Mucci

**Affiliations:** ^1^Department of Psychology, European University of RomeRome, Italy; ^2^Montpellier Business SchoolMontpellier, France; ^3^Department of Experimental and Clinical Medicine, University of FlorenceFlorence, Italy

**Keywords:** expatriate workers, work-related stress, economic stress, fear in the workplace, stress, health promotion, workplace, occupational medicine

## Abstract

Companies’ internationalization appears to be fundamental in the current globalized and competitive environment and seems important not only for organizational success, but also for societal development and sustainability. On one hand, global business increases the demand for managers for international assignment. On the other hand, emergent fears, such as terrorism, seem to be developing around the world, enhancing the risk of expatriates’ potential health problems. The purpose of this paper is to examine the relationships between the emergent concept of fear of expatriation with further workplace fears (economic crisis and dangerous working conditions) and with mental health problems. The study uses a quantitative design. Self-reported data were collected from 265 Italian expatriate workers assigned to both Italian and worldwide projects. Structural equation model analyses showed that fear of expatriation mediates the relationship of mental health with fear of economic crisis and with perceived dangerous working conditions. As expected, in addition to fear, worries of expatriation are also related to further fears. Although, the study is based on self-reports and the cross-sectional study design limits the possibility of making causal inferences, the new constructs introduced add to previous research.

## Introduction

The globalization of markets that has taken place in recent decades was a great opportunity for companies to become known and to operate in a wider context (Biemann and Andresen, 2010; Andresen et al., 2014). This phenomenon led to the possibility that many of the managers who served in a national territory could be transferred to foreign countries, characterized by different cultures and work processes (Sims and Schraeder, 2005).

However, working globally involves changes in occupational dynamics and in the levels of job complexity, and it also requires great skills of adaptation and adjustment (Black et al., 1991; Black and Gregersen, 1999). Most of the researches conducted on adjustment in a foreign country concerned so-called *expatriate workers* or *expatriates* (Sims and Schraeder, 2005), who are those workers sent from their own organization to follow projects or to lead company sectors abroad. An expatriate can be properly defined as one who works in a foreign country for a period of at least 6 months (Birdseye and Hill, 1995; Jones, 2000; Chan et al., 2015). However, shorter forms of expatriation also exist, as shown in the present study.

Supporting expatriates in performing their tasks in a new environment is nowadays essential for companies. Accordingly, researchers have studied expatriated performance and adaptation and evaluated the influence of specific practices of human resource management on their behavior. Relevant dimensions have been identified (Mol et al., 2005; Cheng and Lin, 2009), such as training, to support expatriates in dealing with different cultural values, unexpected behavioral rules, and language barriers. On the other hand, the dark sides of the expatriation experience have also been studied, such as the possible failure of the assignment, leaving without having finished the task, or psychologically withdrawing and performing worse than they usually would. Failure may be particularly expensive in human and monetary terms (Baruch, 2004).

However, adaptation/adjustment may be defined as the comfort degree (or the stress absence) associated with the role of the expatriate (Bhaskar-Shrinivas et al., 2005); expatriates who fail to face the demands of a job and do not properly adapt to a new environment may experience high levels of stress (Perone et al., 2008).

The scenario of stressors among expatriates seems complex, from the micro-environment and the macro-environment to the mega-environment (Lei et al., 2004). In particular, according to Bhaskar-Shrinivas et al. (2005), work assignments to be carried out abroad lead to greater stress when the following situations occur:

(a) when the leader perceives his role as unclear, or rather he does not understand which tasks are actually his and what the company expects from him;
(b) when the leader feels he has a low decision latitude; if he does not feel free to make decisions without first having to ask and obtain the green light from his company;
(c) if the position is considered too demanding, difficult or new; or in a situation that the leader does not feel up to the task of handling because of lack of experience or lack of capacity;
(d) when the manager recognizes that there is a conflict, such as in a case where certain tasks cannot be completed because that would hamper the achievement of other business objectives he is trying to achieve.

Black et al. (1991) observed that expatriates tend to suffer a greater number of relapses after periods of stress. Jones (2000), in a review, identified some risk factors that could not only adversely affect health, but could also lead to developing fear and anxiety of expatriation: risk of being involved in accidents; quality of living conditions; working conditions; risk of disease contagion; fear of being involved in violence; kidnappings and terrorist acts.

These risk factors are analyzed below:

– *Risk of being involved in accidents.* This fear is typically supported by the objective evidence that in some countries there are very low driving standards and poor road safety. Moreover, in some countries the roads are of low quality.
– *Quality of living conditions.* The quality of food and hygiene is one of the most important factors to ensure the adaptation of an expatriate to the new job environment. For example, good water quality cannot be ensured in all countries. Drinking poor quality water could cause the development of oral infections or gastrointestinal problems. The same effects can also be produced by eating non-controlled food. As far as lifestyle is concerned, a lack of leisure activities and difficulties in communication (for example, poor Internet and telephone functionality) may be a concern.
– *Working conditions.* There are higher psychological and physical strains in developing countries, which can inhibit the expatriate’s ability to cope with perceived stress and can eventually increase unsafe practices. Heavy traffic and low control of industrial gas emissions could also affect the health of expatriates. Also, the presence of pristine nature in some working locations might interact negatively with a lower standard of safety and health.
– *Chances of disease contagion.* Expatriates should be informed on the prevalence of diseases in the host country before their trip or during their stay. The possibility of having specific vaccines would be an important protective factor against possible contagion. However, the fear of contagion from some illness might be always present in some countries. Psychological susceptibility to become stressed by the potential contagion also appears important.
– *Fear of being involved in violence, kidnappings, and terrorist acts*. This issue, once confined to few world regions, seems now to be more widespread (Bader and Schuster, 2015).

Given that anxiety could significantly decrease people’s psychological well-being and mental health, there is increasing empirical research on the effect of fear in the workplace (Mueller and Tschan, 2011). Fear, especially if chronic, may damage, in particular, the immune, the nervous and the cardiovascular systems (Shiba et al., 2016).

The human body may be weakened by states of fear, especially if chronic. In particular, the immune, the nervous and the cardiovascular systems are damaged, but equally, the gastrointestinal tract and the reproductive system are not spared. In particular, the fear may compromise the decoding of emotions and decision making processes, making the subject susceptible to intense emotions and impulsive reactions and, consequently, to making inappropriate actions. Fatigue, depression, accelerated aging, and even premature death may be the further consequences of long-term fear (Shiba et al., 2016).

Furthermore, the literature shows that expatriate workers have an increased risk of consuming psychotropic and narcotic substances as well as of abusing of psychotropic drugs (Aubry et al., 2012; Bianchi et al., 2014; Kaeding and Borchers, 2016).

Our study enhanced the literature by being the first to look at a set of important fears among expatriates. In particular, we aimed to find out how the emergence of fear of expatriation, induced by mental health problems, might impact on the expatriate’s further fears in the workplace, using data from a survey of 265 Italian expatriate workers.

Building on the stress perspective (Lazarus and Folkman, 1984; Lazarus, 1991), we have, in particular, examined the following issues: how mental health is associated with fear; the relationship between fear of expatriation and fear of economic crisis as well as perceived dangerous working conditions; the mediation of fear with mental health and the development of further fears.

We intend to conceptualize fear of expatriation due to the risk factors discussed above. Indeed, this study contributes to the literature on expatriates’ health by testing an emergent model for the prevention of mental health issues. This paper proceeds as follows. First of all, we present the conceptual model and the derived hypotheses. Then, we explain the methodology used. Finally, the results and discussion (including limitations and research perspectives) are considered.

### Model Development

**FIGURE 1:**
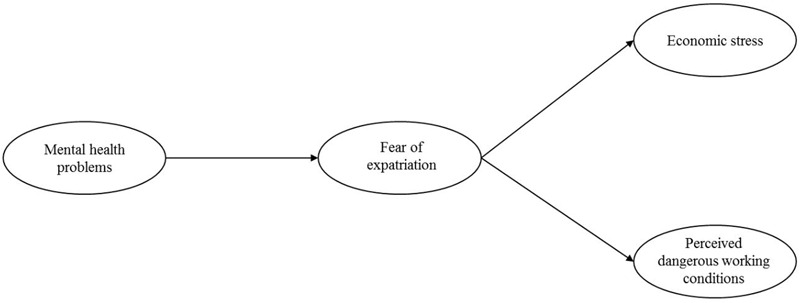
**Conceptual model**.

Expatriate workers often experience difficulties in their adjustment to new work and living situations and, consequently, they are at risk of developing mental health problems (Costa et al., 2015; Zhu et al., 2016). This situation may enhance the fear of violence and of poor living and working conditions during the experience abroad (Lazarus and Folkman, 1984). This fundamental concept is the basis of our conceptual model (**Figure 1**).

With this in mind, workers can be severely traumatized not only by actual violence but also from any potential violence (Bader et al., 2015). For instance, terrorism is quickly spreading (Leistedt, 2013).

Data from the Global Terrorism Database (GTD) of the National Consortium for the Study of Terrorism and Responses to Terrorism (START) (2015) regarding global terrorism shows that in 2014 such attacks relate to 95 countries. In 2014, the worldwide attacks numbered 13,463 (35% more than in 2013), which led to more than 32,700 deaths and more than 34,700 injured people. The geographical distribution is highly concentrated. Sixty percent of these attacks took place in five countries (Iraq, India, Afghanistan, Pakistan, and Nigeria), while 78% of the fatalities caused by terrorism took place in five countries (Iraq, Nigeria, Afghanistan, Pakistan, and Syria). The strategy of the most developed terroristic groups – e.g., *Islamic State of Iraqi and the Levant* (ISIL) and *Boko Haram* – not only provides for violent attacks on military or civilian points, but also for kidnapping, torture, and rape. These practices increase the fear of the people – particularly those who are located in the directly involved geographical areas – and of international public opinion. Workers with mental health problems might be particularly vulnerable to developing fears of these practices. The use of social media by ISIL has allowed for the extreme visibility of this organization with a widespread dissemination of its terroristic contents (United States Department of State Publication Bureau of Counterterrorism, 2015) and might increase anxiety in workers with pre-existent mental health problems or stress (Solberg et al., 2015; Glad et al., 2016; Paz García-Vera et al., 2016).

The context of living and working conditions in the host country is another factor associated with expatriates’ psychological well-being. Frazee (1998) pointed out that healthcare is one of the main issues for expatriates: more than one-third of international assignments are dissatisfied with the health assistance they receive.

The standard of healthcare around the world varies in a very important way. However, discrepancies may exist even among different regions of the same country. In addition, expatriates might be afraid of not receiving an adequate and timely treatment for all types of injury and, moreover, the sanitary conditions might not be good, increasing the risks of contagions or illness. These concerns affect virtually all expatriate workers, but may result in real states of fear in subjects with mental health problems and may generate the acute and chronic worsening of any already existing clinical situation (Pierre et al., 2013; Cleary et al., 2014; Wilde and Gollogly, 2014).

*Hypothesis 1:* Mental health problems generate fear of expatriation.

The second part of the model is focused on the development of further fears in the workplace.

Despite the numerous relevant stressors in global assignments, in our conceptual framework we mainly focused on two areas of fear of expatriation. The first is related to violence, intended both as physical and psychological. The second is related to the perceived impeded living and working conditions (including workplace safety, illness contagion, and lifestyle).

As already explained, the presence of a pre-existing state of fear or anxiety may enhance the likelihood of negative stimulus to elicit fear. Indeed, emotions are specific to the context and imply a person-environment relationship. More specifically, emotions embody a particular theme, reflecting the way the individual sees his/her relationship with the environment in a given situation (Lazarus, 2001).

The fear of expatriation might negatively influence the perception of the safety environment and the anxiety caused by economic crisis. Moreover, expatriates are often exported to societies with weaker and less expensive H&S policies (Heymann, 2003), raising a perception of unsafe working conditions (Curcuruto et al., 2015).

In our model we expect a mediation process of fear of expatriation among mental health and further fears in the workplace. First, mental health problems generate anxiety and fear. Fear can impair the formation of long-term memories and can cause damage to certain parts of the brain, such as the hippocampus (Besnard and Sahay, 2016). This can make it even more difficult to regulate fear and can leave a person anxious most of the time. The threats to our security impact our mental well-being, whether they are real or perceived, generating multiple fears.

*Hypothesis 2:* Mental health problems, through the mediation of fear of expatriation, influence further fears in the workplace: dangerous working conditions and economic stress.

## Materials and Methods

### Procedure and Participants

This study was conducted in a large international company dealing with technology and services in heavy industry. The expatriate managers employed in this company were all invited to participate in the study. Expatriation services in this company are usually in short form. Expatriates spend cyclically 28 days outside the workplace (often in platforms or yards located worldwide). The final respondents were 265 employees (response rate = 70%) working in multiple locations (Italy, Europe, Middle East, Asia, Africa, Australia, etc.).

The survey was administered through the corporate Intranet, ensuring anonymity, and privacy rules. A video, in which an industrial psychologist and an occupational physician explained the procedure of questionnaire compilation and the survey aims, was also made available through the corporate Intranet.

The sample consisted of only men in managerial positions. Workers were, on average, relatively young: 18.9% 30 years old or younger, 48.3% from 31 to 40 years old, 23.4% from 41 to 50 years old, and only 9.4% were over 50. Regarding job tenure, 23.8% of the participants had worked from 0 to 5 years, 30.9% of participants had worked from 6 to 10 years, 30.9% of the participants had worked from 11 to 20 years, and 14.3% of participants more than 20 years. Finally, the majority of employees had long working hours (17.4% 50 h per week, 26.6% 50–60 h per week, 56% more than 60 h per week).

### Measures

After collecting some socio-demographic variables, participants completed the scales on fear of expatriation, economic stress, dangerous working conditions and psychological distress. The scales used in this study are described below.

#### Fear of Expatriation

Fear of expatriation was measured by a new questionnaire, developed by our research group and called *Fear of expatriation scale* (Supplementary Material).

The measure is composed of two dimensions:

(a) *fear of violence/terrorism* – (two items) employees are scared of being subjected to violence/terrorism (e.g., “I am scared of being the object of physical violence – kidnapping, terrorism, etc.”);
(b) *fear of the working and living conditions* – (three items) employees are worried about the working and living conditions and about healthcare (e.g., “I am scared of contracting a disease”).

The scores were collected, for each dimension, through a five-point Likert scale (from 1: “strongly disagree” to 5: “strongly agree”). As this instrument was developed for this research, we evaluated the construct validity and reliability of the fear of expatriation scale in order to investigate its psychometric properties. We assessed the construct validity (convergent validity and discriminant validity) of the scale by conducting a confirmatory factor analysis (CFA) in order to compare the hypothesized factorial model involving two distinct factors – fear of violence/terrorism and fear of the working and living conditions – with a one-factor model. Results showed that the hypothesized two-factor model yielded a good fit to the data (χ^2^[4] = 9.35, *ns*; CFI = 0.99; RMSEA = 0.07; SRMR = 0.02) and outperformed that of the one-factor solution (χ^2^[4] = 20.25, *p* < 0.01; CFI = 0.97; RMSEA = 0.11; SRMR = 0.02; Δχ^2^(1) = 10.90, *p* < 0.01), thus supporting the distinctiveness between the two sub-dimensions of fear of violence/terrorism and fear of the working and living conditions. Furthermore, standardized regression coefficients of items on each factor were all higher than 0.50 (Hair et al., 2007), thus supporting the convergent validity of the factors (range = 0.62–0.85). However, CFA results also indicated that the correlation among latent constructs was higher than 0.89. This therefore suggests that the two dimensions might be best combined on an overall scale of fear of expatriation (Kline, 2011). Accordingly, in our subsequent analyses to test Hypotheses 1 and 2, we considered only the overarching fear of expatriation scale, and not its separate dimensions. Finally, internal consistency, which was assessed by the calculation of reliability coefficients (Cronbach’s alpha), was 0.86, 0.76, and 0.80 for the overall fear of expatriation scale, the fear of violence/terrorism dimension and the fear of the working and living conditions dimension, respectively. Thus, this indicated good internal consistency of the measure (Nunnally, 1978).

#### Economic Stress

Economic stress was measured with the scale about subjective economic stress included in the recent *Stress Questionnaire* (SQ), developed and validated in Italy (Giorgi et al., 2013; Mucci et al., 2015). The economic stress measure is composed of two dimensions:

(a) *fear of the economic crisis* (five items) – employees perceive that the organization is suffering from the economic crisis (e.g., “I am scared that my organization is affected by the economic crisis; I am scared that my organization, due to the economic crisis, will be subjected to downsizing”);
(b) *non-employability* (five items) – employees perceive that their working competencies would not permit them to acquire another job in the market (Caricati et al., 2016) [e.g., “My professionalism is not spendable (recognized) in the labor market; My staying in the organization is linked to the difficulty of outplacement in the labor market”].

Each dimension includes five items in a five-point Likert scale (from 1: “strongly disagree” to 5: “strongly agree”).

#### Dangerous Working Conditions

We used a scale, included in the above mentioned *Stress Questionnaire* (Giorgi et al., 2013; Mucci et al., 2015), that covers two factors in a five-point Likert scale (from 1: “strongly disagree” to 5: “strongly agree”):

(a) *dangerous working conditions* (four items) – this measures the extent to which the organization’s working conditions are dangerous for employees’ H&S (e.g., “Working methods are dangerous for my own and employees’ health; The ergonomics of my work are harmful for my health”);
(b) *working conditions* (five items) this measures the lack of general working conditions in the workplace, e.g., “Risk of accidents is low in my workplace (*reverse scored*); My workplace is free of workplace health hazards, chemical gas, smoke, etc. (*reverse scored*).”

#### Psychological Distress

This was measured with the General Health Questionnaire (GHQ-12; Goldberg and Hillier, 1979; Fraccaroli et al., 1991). The scale asks whether the respondent has experienced a particular symptom or behavior related to general psychological health recently. Each item is rated on a four-point Likert-type scale (0-1-2-3). A higher score indicates a greater degree of psychological distress. In this study we particularly focus on the sub-dimension “anxiety and insomnia” (seven items, e.g., “Considering the last few weeks, have you recently […] felt constantly under strain?”).

## Results

Following Anderson and Gerbing’s (1988) two-step structural equation modeling (SEM) procedure, we tested a measurement model (CFA) by determining whether each measure’s estimated loading on its expected underlying factor was significant. This allowed us to establish discriminant validity among the study constructs. Then, a structural model was performed to estimate the fit to the data of the hypothesized model in which fear of expatriation mediates the relationship of mental health problems with economic stress and perceived dangerous working conditions (Hypotheses 1 and 2). A CFA was, therefore, performed with *Mplus, version 7.11* (Muthén and Muthén, 1998–2010), with the four variables measuring mental health problems, fear of expatriation, economic stress, and perceived dangerous working conditions. Moreover, the variables’ dimensions were used as indicators of their corresponding latent constructs in the measurement and structural models. These dimensions were formed by averaging the items of each sub-scale for the four latent variables. We therefore obtained three indicators for mental health problems, two indicators for fear of expatriation, two indicators for economic stress, and two indicators for perceived dangerous working conditions.

To evaluate the model fit, we considered chi-square (the higher the values are, the worse is the model’s correspondence to the data), and used both absolute and incremental fit indexes. Absolute fit indexes evaluate how well an *a priori* model reproduces the sample data. In our study, we focused on three absolute fit indexes: the standardized root mean square residual (SRMR), for which values of less than 0.08 are favorable, and the root-mean-square error of approximation (RMSEA), which should not exceed 0.10 (Browne and Cudeck, 1993; Kline, 2011). Incremental fit indexes measure the proportionate amount of improvement in fit when a target model is compared with a more restricted, nested baseline model (Schreiber et al., 2006). We considered the comparative fit index (CFI), for which values of 0.90 or greater are recommended (Schreiber et al., 2006).

As expected, the hypothesized four-factor model yielded a good fit to the data: χ^2^(20) = 61.17, CFI = 0.95, RMSEA = 0.09, SRMR = 0.05 (**Table 1**). Additionally, as shown in **Table 2**, this model had a significantly better fit than alternative, more parsimonious models (*p* < 0.01), supporting the distinctiveness of the study variables. **Table 2** displays the descriptive statistics, correlations, and reliability coefficients of the variables.

**Table 1:** Fit indices for confirmatory factor analyses.

Model	χ^2^	*df*	Δχ^2^	Δ *df*	CFI	RMSEA	SRMR
Hypothesized four-factor model	61.175^∗^	20	–	–	0.95	0.09	0.05
Three-factor models							
Combining MHP and FEX	128.08^∗^	23	^∗^	3	0.87	0.13	0.07
Combining MHP and FEC	94.25^∗^	23	^∗^	3	0.91	0.11	0.07
Combining MHP and DWC	185.43^∗^	23	^∗^	3	0.80	0.16	0.10
Combining FEX and FEC	153.02^∗^	23	^∗^	3	0.84	0.15	0.07
Combining FEX and DWC	184.11^∗^	23	^∗^	3	0.80	0.16	0.10
Combining FEC and DWC	274.90^∗^	23	^∗^	3	0.69	0.20	0.18
Two-factor model							
Combining MHP, FEX and FEC	159.29^∗^	25	^∗^	5	0.83	0.14	0.07
Combining MHP, FEX and DWC	247.55^∗^	25	^∗^	5	0.73	0.18	0.10
Combining MHP, FEC, and DWC	211.30^∗^	25	^∗^	5	0.77	0.17	0.10
Combining FEX, FEC, and DWC	263.09^∗^	25	^∗^	5	0.71	0.19	0.11
One-factor model	269.46^∗^	26	^∗^	6	0.70	0.19	0.10

*N = 265. CFI, comparative fit index; RMSEA, root-mean-square error of approximation; SRMR, standardized root mean square residual; Δχ^2^, chi-square difference tests between the best-fitting model (Hypothesize four-factor model) and alternative models. MHP, mental health problems; FEX, fear of expatriation; FEC, fear of economic crisis; DWC, perceived dangerous working conditions. ^∗^*p* < 0.01.*

**Table 2:** Descriptive statistics and correlations.

Variables	*M*	*SD*	1	2	3	4	5	6
(1) Age	-	-	-					
(2) Organizational tenure	-	-	0.57^∗∗^	-				
(3) Mental health problems	10.60	5.09	-0.11	-0.05	(0.80)			
(4) Fear of expatriation	7.05	2.33	-0.22^∗∗^	-0.02	0.52^∗∗^	(0.86)		
(5) Fear of economic crisis	2.55	0.55	-0.02	0.00	0.36^∗∗^	0.29^∗∗^	(0.84)	
(6) Perceived dangerous working conditions	3.37	0.68	-0.22^∗∗^	0.04	0.34^∗∗^	0.57^∗∗^	0.19^∗∗^	(0.84)

*N = 265. Internal Consistency coefficients (Cronbach’s alphas) appear along the diagonal in parentheses. ^∗∗^*p* < 0.01.*

In order to examine the hypothesized model, we performed SEM with Mplus. SEM offers the following advantages:

(a) controlling for measurement errors when the relationships among variables are analyzed (Hoyle and Smith, 1994);
(b) comparing the goodness-of-fit of the hypothesized model with other alternative models (Cheung and Lau, 2008).

Thus, we tested our proposed structural model and compared it with alternative models. Additionally, when conducting SEM analyses, we controlled for the effects of age and organizational tenure on both the mediator and the dependent variables. Fit indexes for each tested model are presented in **Table 3**.

**Table 3:** Fit indices for nested structural models.

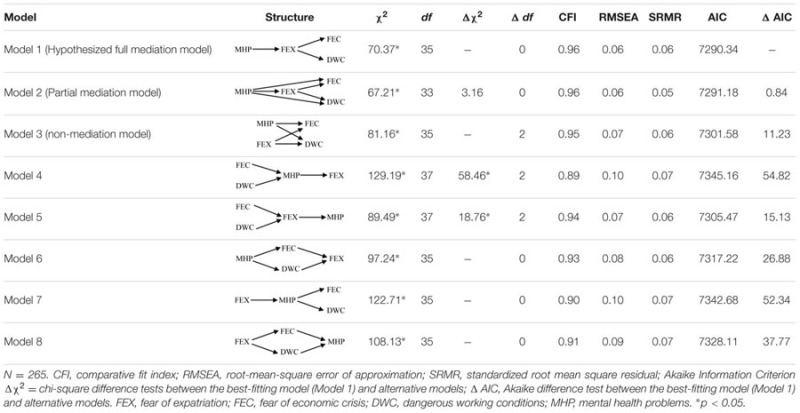

**FIGURE 2:**
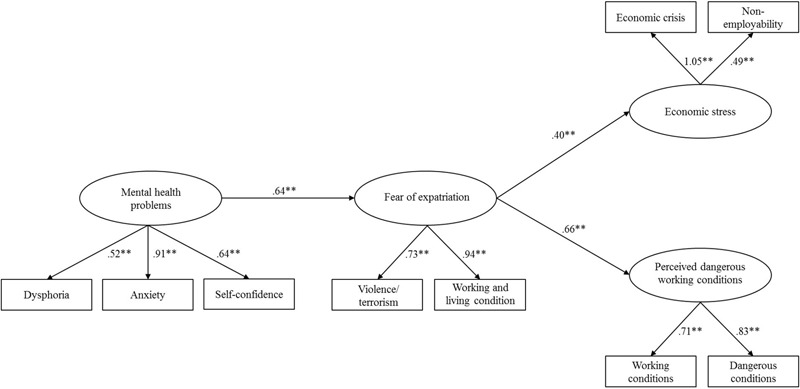
**Completely standardized path coefficients for Model 1.** ^∗∗^*p* < 0.01.

The hypothesized model (Model 1), which is a full mediation model, displayed a good fit to the data: χ^2^(35) = 70.37, CFI = 0.96; RMSEA = 0.06; SRMR = 0.06. Specific inspection of direct relationships further revealed that mental health problems were positively associated with fear of expatriation (β = 0.64, *p* < 0.01), thus supporting Hypothesis 1. Additionally, fear of expatriation, in turn, was positively related to economic stress (β = 0.40, *p* < 0.01) and perceived dangerous working conditions (β = 0.66, *p* < 0.01), thus providing preliminary support for Hypothesis 2. Completely standardized path coefficients for Model 1 are depicted in **Figure 2**.

To assess whether the hypothesized model was the best representation of the data, we then compared its fit to that of different alternative models. First, we assessed a partial mediation model, which included two additional direct paths from mental health problems to economic stress and perceived dangerous working conditions. This model yielded an adequate fit to the data (χ^2^[33] = 67.21, CFI = 0.96; RMSEA = 0.06; SRMR = 0.05), but it was not significantly better than Model 1, as revealed by the chi-square difference (Δχ^2^[1] = 3.16, *ns*). Moreover, the additional direct relationships of workplace mental problems with economic stress (β = 0.13, *ns*) and dangerous working conditions were not significant (β = 0.03, *ns*).

Next, we compared the hypothesized model with a non-mediation model (Model 3), which only included direct paths from mental health problems and fear of expatriation to economic stress and perceived dangerous working conditions. Results revealed that the non-mediation model was a slightly worse fit to the data than the hypothesized fully-mediated model (χ^2^[35] = 81.61, CFI = 0.95; RMSEA = 0.07; SRMR = 0.06). However, because this model had the same degrees of freedom as the hypothesized model, the statistical significance of the chi-square difference could not be calculated. Accordingly, we used the Akaike Information Criterion (AIC), instead of the chi-square, to compare the two models. The hypothesized model is considered to be superior to the non-mediation model if the former has an AIC value lower than the latter by four or more units (Burnham and Anderson, 2002). Results revealed that Model 3 had an AIC of 7301.58 compared to an AIC of 7290.35 for Model 1, suggesting that the hypothesized full mediation model represents a superior fit to the data than the non-mediation model (ΔAIC = 11.23).

Furthermore, because mental health problems, fear of expatriation, economic stress, and perceived dangerous working conditions were all measured at the same time, reverse relationships could also be expected between the four variables. In order to rule out this possibility, we therefore compared the hypothesized model against a set of alternative models that specified all the possible reverse indirect relationships among the study variables, namely: the indirect relationship of economic stress and perceived dangerous working conditions with fear of expatriation via mental health problems (Model 4); the indirect relationship of economic stress and perceived dangerous working conditions with mental health problems via fear of expatriation (Model 5); the indirect relationship between mental health problems and fear of expatriation via economic stress and perceived dangerous working conditions (Model 6); the indirect relationship of fear of expatriation with economic stress and perceived dangerous working conditions via mental health problems (Model 7); the relationship between fear of expatriation and mental health problems via economic stress and perceived dangerous working conditions (Model 8). Again, because Models 6–8 had the same degrees of freedom as the hypothesized model, we compared the model fit by using the AIC difference test. As can be seen from **Table 3**, Models 4–8 all yielded a worse fit to the data than the hypothesized model. Overall, results from model comparison suggested that Model 1 was the best fitting model. We therefore retained the hypothesized fully-mediated model.

Finally, in order to assess whether the indirect relationship of mental health problems with economic stress and perceived dangerous working conditions through fear of expatriation was significant (Hypothesis 2), we calculated 95% bootstrapping confidence intervals (Preacher and Hayes, 2008; Preacher and Kelley, 2011). Based on 5,000 bootstrap replications, results indicated that mental health problems had an indirect positive effect on economic stress (indirect effect = 0.18; 95% CI = 0.12, 0.24) and perceived dangerous working conditions (indirect effect = 0.18; 95% CI = 0.12, 0.24) via fear of expatriation. Hypothesis 2 was therefore fully supported.

## Discussion

In a globalized working environment with turbulence in the economy and in the security expatriate workers are confronted with several stressors, making international assignments potentially stressful. Accordingly, expatriate managers might report lower psychological well-being and anxiety (Wang and Kanungo, 2004; Shaffer et al., 2006; Wang and Nayir, 2006).

At the same time, fears are now increasing in the workplace, marked by emotional discomfort, apprehension, or concerns about the internal and external environment, and expatriates seem particularly at risk. These symptoms can progress to more severe psychosomatic symptoms, including further anxiety and additional fears (Blum et al., 2010; Besnard and Sahay, 2016).

In our model, fear of expatriation is particularly associated with mental health problems. This result is in line with the field literature (i.e., Perone et al., 2008; Andresen et al., 2014; Aracı, 2015). In addition, an expatriate might go through several personal and professional problems. In fact, expatriation is associated with a lot of unhealthy issues such as stress, anxiety, loneliness and homesickness, generating a sort of potential and prolonged *cultural shock* (Barón et al., 2014). Finally, it must be emphasized that expatriates cannot count on the support of family and/or other trusted people if they need it (Bhaskar-Shrinivas et al., 2005).

Fear of expatriation might generate a spiraling effect in which people, feeling more anxious, might become less engaged in the workplace, developing beliefs that money and extrinsic reward are the most important aspects of employment (Gerhart and Fang, 2015). At the same time, they may be more worried about their financial situation and their ability to hold on to their jobs and their benefits, developing their fear of economic crisis.

On one hand, from a subjective point of view, H&S measures – under certain circumstances (for instance, the emergence of fear in the workplace) – do not necessarily express feelings of safety, but rather can be interpreted as latent danger (Bader and Berg, 2012) generating a widespread anxiety. On the other hand, from an objective point of view, expatriates are often *exported* to societies with weaker and less expensive H&S policies and less organized labor forces, bringing potentially stressful and unsafe working conditions (Heymann, 2003) to expatriates. This might raise a perception of dangerous working conditions. Moreover, H&S procedures are usually highly specific to each country and with very important differences – related to the various national legislations and traditions – and working conditions abroad are generally perceived as less familiar and presenting higher risk (Taylor et al., 2007).

Expatriates are a group of people with a high cumulative risk of exposure to illness and injury (including the increased risk of certain vaccine-preventable illnesses) due to changes in travel patterns and activities, lifestyle alterations, and increased interaction with local populations. Pre-travel immunization management provides one safe and reliable method of preventing infectious illness in this group; however, this might not be enough to cope with anxiety (Vaid et al., 2013; Shepherd and Shoff, 2014). In addition, there are diseases that are not preventable with vaccines. These diseases, in particular of a viral nature, seem to spread faster nowadays – such as, for example, the recent Ebola or Middle East Respiratory Syndrome (MERS) outbreaks – and might be frightening for expatriates (Cohen et al., 2016; Regan et al., 2016).

In summary, the non-optimal H&S perception (Beeley et al., 1993), the risk of contracting infectious diseases (Jones, 1999; Hamlyn et al., 2007) and the unsuitability of medical care (Pierre et al., 2013) were also evaluated under the construct of the fear of expatriation.

In our study, expatriates reported being frightened by the risk of becoming involved in accidents during their frequent moves from one country to another. In particular, this fear seemed to be greater for traffic accidents (Wilks et al., 1999), but a fear of flying was also described (Hack-Polay, 2012).

A further and significant stress factor for expatriate workers is the managers’ fear of terrorist attacks or other fatal events involving the governments of countries where there are companies’ headquarters (Leistedt, 2013; Bader and Schuster, 2015). Finally, in our model we consider the concerns about the working and living conditions (Costa et al., 2015; Zhu et al., 2016).

In addition, our model has shown that the presence of fear of expatriation may, in turn, generate further fears in the workplace. In particular, fears of both the economic crisis and of the foreign working conditions are mediated by fear of expatriation. In fact, fear could adversely disturb human thinking and decision-making processes, leaving the individual more susceptible to generating further fears, as in a vicious circle (Barón et al., 2014; Besnard and Sahay, 2016).

Our findings are in line with the basic propositions of Lazarus and the “affective events theory” (AET; Weiss and Cropanzano, 1996). The latter pointed out that an emotion experienced by a worker (e.g., fear) may impact on later within-person emotions (e.g., fear again), influencing different organizational outcomes.

As far as economic crisis is concerned, economic stress might be more frightening for those who have more invested in the company, such as expatriates. In fact, these workers, being away from home for extended periods of time, are completely absorbed by their job and, therefore, they may be an easier target for contagious negative emotional cycles (Quantin et al., 2012). Moreover, they could be most affected by the psychological impacts of the economic crisis and its consequences as they have lower levels of social support (Fernandez et al., 2015).

Similarly, fear of expatriation may significantly lead to perceiving *a priori* all foreign working conditions as being more dangerous. These findings support our assumption and the literature (Perone et al., 2008; Andresen et al., 2014). The impact of working and living conditions – resulting, for instance, in perceived risks of harms – might be higher if expatriates are scared by the impeded living conditions or by the threat of violence. This might have several negative implications as often expatriates have the tendency to “get the job done” as smoothly as possible, so they can return home again (Aracı, 2015). However, this specific issue should be investigated in future studies.

Our findings provide interesting contributions both to the literature and to the managerial practices in the field of foreign work.

First, we believe that the subjects in the process of leaving their own country should be mentally healthy and not feeling frightened by either the place of destination or the assigned tasks. According to Lazarus and Folkman (1984), if an expatriate is worried and anxious, it is less likely that he/she will ever adjust. Therefore, it is essential to help expatriates to prevent the development of any type of fear. Strategies of prevention and rationalization, particularly useful in this sense, can be implemented through several instruments: specific training for foreign services, company’s reference facilities in countries where employees come to work, remote counseling (on-line/phone) provided by occupational physicians and psychologists affiliated with the company, company procedures for immediate repatriation in the case of adverse events (e.g., terrorism, infective outbreaks, and health problems), etc.

In the second place, stress management training is also recommended. From this perspective, issue-focused coping strategies seem crucial to counteract fearful feelings as well as to minimize the states of anxiety and distress (Folkman et al., 1986).

Thirdly, companies should conduct preventive screening to identify the human resources to be sent to foreign countries, favoring those who have demonstrated both a highly qualified professionalism in their field and robust mental health (Stone, 1991; Di Fabio, 2014, 2015).

Consequently, a process for the successful management of expatriation needs to be adopted using both individual and organizational strategies to reduce the possibilities of stress among expatriates. At the organizational level, selection, training, healthcare activities, and counseling need to be implemented and monitored in order to prevent the diffusions of workplace fears. At the individual level, expatriates should be psychologically supported, e.g., with mentoring and coaching, analyzing competencies, health perceptions, and mood over time (De Paul and Bikos, 2015).

Our study has many and innovative strengths but is not without limitations. Although the expatriates worked worldwide, the sample was limited to a single company, limiting the generalizability of the results. In addition, the sample was composed only of men. However, by virtue of family demands, men expatriate much more frequently than women. Our scale *fear of the expatriation* is new in the literature and, consequently, a replication of this study is needed. In addition, we look forward to more large studies whose starting points are the results of the first application of our scale. In particular, comparative research evaluating stress responses between Italian and other ethnic populations around the world would be particularly helpful.

This study used a cross-sectional design, resulting in the impossibility of determining causal relationships. Longitudinal research is needed in order to provide further evidence that mental health problems cause fear of expatriation, which, in turn, may generate additional workplace fears.

In summary, following this new research path, we have developed a new model, formulated a new theory that found an association between mental health and fears in the workplace, and explored different fears in the workplace and their links.

Our results confirmed our innovative hypothesis and we suggest that companies’ key people take into account the construct of fear of expatriation for business health purposes. With this in mind, companies need proper advice from qualified consultants such as occupational physicians and industrial psychologists. An investment dedicated to the prevention and protection of the H&S of expatriate workers is not only an instrument of risk assessment – in the context of the obligations under the laws of EU countries in the field (e.g., the Italian Legislative Decree n. 81 and subsequent amendments) – but also, and moreover, a significant tool to improve the company’s business.

Some studies have estimated that the cost associated with the failure of expatriation would be about one million USD (Insch and Daniels, 2002; Wentland, 2003). Overall, considering the aggregate data about the American situation, Punnett (1997) has calculated that US companies spend a total of up to two billion USD annually to address the failures of their expatriate managers. In such a context, it is legitimate to expect that intervention strategies – such as a careful selection of personnel to be devoted to foreign missions and the development of actions aimed at improving the real and perceived well-being in destination countries – will lead to significant *returns on investment* (ROI) for the companies.

## Author Contributions

GG, FM, JF-P, GA, and NM equally contributed to all the following issues of the research: conception and design of the work; acquisition, analysis, or interpretation of data for the work; drafting the work and critically revising it; final approval of the version to be published; agreement to be accountable for all aspects of the work in ensuring that questions related to the accuracy or integrity of any part of the work are appropriately investigated and resolved.

## Conflict of Interest Statement

The authors declare that the research was conducted in the absence of any commercial or financial relationships that could be construed as a potential conflict of interest.
