# Supplementary material for: Expatriates’ Multiple Fears, from Terrorism to Working Conditions: Development of a Model
Source: Front Psychol. 2016 Oct 13;7:1571. doi: 10.3389/fpsyg.2016.01571 (PMC5062027; doi:10.3389/fpsyg.2016.01571)
Supplement: Supplementary file 1 [file Data_Sheet_1.docx]

**APPENDIX**

**TABLE A1 | Fear of expatriation scale**

**Nothing at all - Extremely (Likert 1-5)**

The original scale is in Italian. In this appendix we present translation of the scale in American English.

We investigated the adjustment to the working conditions of your foreign assignment. Are you scared of the risk of:

- Incidents (car, boat, plane, etc.)
- Being subject of physical violence (kidnapping, terrorism, etc.)
- Having health hardships due to bad working conditions (noise, poor lightness, etc.)
- Being sick due to the new lifestyle
- Contracting a disease
